# An integrated approach based on advanced CTG parameters and Doppler measurements for late growth restriction management

**DOI:** 10.1186/s12884-021-04235-0

**Published:** 2021-11-16

**Authors:** Giuseppina Esposito, Nicolò Pini, Salvatore Tagliaferri, Marta Campanile, Fulvio Zullo, Giovanni Magenes, Giuseppe Maria Maruotti, Maria Gabriella Signorini

**Affiliations:** 1 Department of Obstetrical-Gynaecological and Urological Science and Reproductive Medicine of the Federico II University, Naples, Italy; 2 Dipartimento di Elettronica, Informazione e Bioingegneria (DEIB), Politecnico di Milano, Milan, Italy; 3 Department of Psychiatry, Columbia University Irving Medical Center, New York, NY USA; 4 Department of Electrical, Computer and Biomedical Engineering, University of Pavia, Pavia, Italy

**Keywords:** Late fetal growth restriction, Doppler ultrasound data, Antepartum fetal heart rate monitoring, Computerized cardiotocography, Phase-rectified signal average

## Abstract

**Background:**

The clinical diagnosis of late Fetal Growth Restriction (FGR) involves the integration of Doppler ultrasound data and Fetal Heart Rate (FHR) monitoring through computer assisted computerized cardiotocography (cCTG). The aim of the study was to evaluate the diagnostic power of combined Doppler and cCTG parameters by contrasting late FGR –and healthy controls.

**Methods:**

The study was conducted from January 2018 to May 2020. Only pregnant women who had the last Doppler measurement obtained within 1 week before delivery and cCTG performed within 24 h before delivery were included in the study. Two hundred forty-nine pregnant women fulfilling the inclusion criteria were enrolled in the study; 95 were confirmed as late FGR and 154 were included in the control group.

**Results:**

Among the extracted cCTG parameters, Delta Index, Short Term Variability (STV), Long Term Variability (LTV), Acceleration and Deceleration Phase Rectified Slope (APRS, DPRS) values were lower in the late FGR participants compared to the control group. In the FGR cohort, Delta, STV, APRS, and DPRS were found different when stratifying by MCA_PI (MCA_PI <5th centile or > 5th centile). STV and DPRS were the only parameters to be found different when stratifying by (UA_PI >95th centile or UA_PI <95th centile). Additionally, we measured the predictive power of cCTG parameters toward the identification of associated Doppler measures using figures of merit extracted from ROC curves.

The AUC of ROC curves were accurate for STV (0,70), Delta (0,68), APRS (0,65) and DPRS (0,71) when UA_PI values were > 95th centile while, the accuracy attributable to the prediction of MCA_PI was 0.76, 0.77, 0.73, and 0.76 for STV, Delta, APRS, and DPRS, respectively.

An association of UA_PI>95th centile and MCA_PI<5th centile with higher risk for NICU admission, was observed, while CPR < 5th centile resulted not associated with any perinatal outcome. Values of STV, Delta, APRS, DPRS were significantly lower for FGR neonates admitted to NICU, compared with the uncomplicated FGR cohort.

**Conclusions:**

The results of this study show the contribution of advanced cCTG parameters and fetal Doppler to the identification of late FGR and the association of those parameters with the risk for NICU admission.

**Trial registration:**

Retrospectively registered.

## Background

Fetal growth restriction (FGR), also known as intrauterine growth restriction (IUGR) is a common complication of pregnancy that has been associated with a variety of adverse perinatal outcomes [1].

The prevalence is estimated to affect approximately 3–9% of all pregnancies, according to different definition used [2, 3].

The key issue in the management of a pregnancy complicated by FGR is the timely identification of the fetus at greatest risk for adverse outcome. FGR evolves from a preclinical phase to clinically apparent growth delay and may ultimately progress to fetal deterioration. Although potential abnormalities in placental development may arise over the continuum of gestational age, common opinion distinguished the early-onset presenting before 32 weeks from the late-onset FGR presenting after 32 weeks [4, 5].

Third trimester obstetrical ultrasound policies differ from country to country, usually women are not routinely scanned in late pregnancy but are selected for third trimester ultrasonography on the basis of pre-pregnancy risk factors, development of obstetric complications, and serial measurement of symphyseal-fundal height. This approach identifies a third of FGR infants or fewer and unidentified FGR is a common finding in perinatal deaths [6]. The late-onset FGR condition represents the 70–80% of FGR cases. It is frequently associated with mild placental perfusion and moderate fetal Doppler Pulsatility Index (PI) abnormalities. Sometimes, it is possible to recognize a chronological evolution of fetal Doppler abnormalities with Umbilical Artery (UA) PI increase, Middle Cerebral Artery (MCA) PI reduction or a co-occurrence of both events [7]. Clinical evolution of fetal deterioration may include a reduction in amniotic fluid volume and non-reassuring cardiotocography (CTG) [4, 5].

Integrated fetal testing is fundamental to detect significant variations in the clinical evolution of late-onset FGR, to accurate schedule fetal surveillance and to make decisions on delivery timing.

Diagnosis of late FGR involves the integration of Doppler ultrasound data and Fetal Heart Rate (FHR) monitoring through computer assisted (or computerized) CardioTocoGraphy (cCTG The cCTG represents a quantitative technological approach of classical visual inspection CTG, by which FHR and Toco parameters can be extracted through computer analysis: it has largely been employed for fetal well-being assessment during the antenatal period [8], especially in pregnancies complicated by FGR [5, 9, 10].

If not diagnosed, late FGR may undergo rapid deterioration leading to severe injury or perinatal death without observable late-stage signs. This might be explained by a combination of causes, which could include the low tolerance of term fetuses to hypoxia and episodes of rapid placental function failure. No important differences in adverse outcomes between induction of labor and expectant monitoring in women with suspected intrauterine growth restriction at term was found, but patients who are keen on non-intervention must have intensive maternal and fetal monitoring [11], therefore, in late FGR the most challenging issue is the timely and adequate diagnosis [12].

The low diagnostic rate of late FGR contributes significantly to pregnancy stillbirths. The unmet need of prenatal surveillance is to identify fetus at risk of late FGR, to improve their management and consequently to plan their delivery before adverse events occur [13].

The aim of the study is to verify if an association between Doppler abnormalities and a set of cCTG parameters exists in late-onset FGR. The same set of cCTG parameters demonstrated its usefulness in the identification of FGRs (irrespective of pathology onset) [14]. For this reason, the contribution of advanced cCTG parameters towards late FGR identification is the focus of this work.

As a secondary aim, we evaluated the role of Doppler and cCTG parameters in identifying the late FGR neonates at higher risk of Neonatal Intensive Care Unit admission.

## Methods

The retrospective study described in this work was carried out at the Department of Obstetrical-Gynaecological and Urological Science and Reproductive Medicine of the Federico II University in Naples (Italy) from January 2018 to May 2020, in collaboration with biomedical engineers of Politecnico di Milano and University of Pavia (Italy). All pregnant women were followed-up, admitted and gave birth in the same Obstetric Unit.

The subset analyzed in this work was obtained by sampling a larger prospective study on a population of 2650 women with control group and pregnancies complicated by diverse etiologies.

Pregnant women were enrolled starting from the 32th week of gestation until end of gestation. Inclusion criteria were Caucasian ethnicity; singleton pregnancy; certain pregnancy dating, calculated from the first day of the last menstrual period and confirmed by ultrasound measurements, according to the population nomograms. Diagnostic criteria for late onset FGR were an AC and/or EFW < 10th centile and at least Doppler UA-PI >95th centile or abnormal MCA <5th centile [15]. As regards delivery, only pregnant women whose elective or urgent delivery occurred for fetal indication as late Doppler changes or pathological CTG were considered in the FGR group, excluding maternal or gestational indications.

We defined a fetus as control group when its growth was appropriate for gestational age without any chromosomal and major congenital anomalies. Control group fetuses were monitored with Doppler and cCTG monitoring at the same gestational weeks of late onset FGR.

Neonatal data such as sex, birthweight, Apgar score, malformation at birth, access to neonatal intensive care, and umbilical artery blood gas values were also collected for both groups.

From the selected population, exclusion criteria were pregestational and gestational diabetes, hypertensive disorders, autoimmune diseases, BMI ≥ 35, fetus with chromosomal and major congenital anomalies, cCTGs with a duration less than 1 h and signal loss greater than 15% through the duration of the recording, and missing neonatal outcome data.

Only pregnant women who had the last Doppler measurement obtained within 1 week before delivery and cCTG performed within 24 h before delivery were included in the study.

**Fig. 1:**
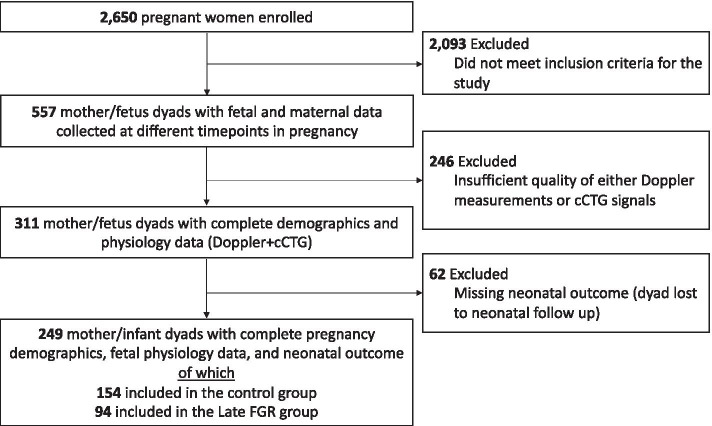
Case/Control Recruitment flow chart

Following the application of the presented selection procedure, 2093 pregnant women did not meet the inclusion criteria, 246 pregnant women had missing data, 62 were lost to neonatal follow-up. Finally, 249 pregnant women fulfilled the inclusion criteria. Specifically, the analyzed cohort comprises of participant with complete information on gestational, fetal and neonatal outcome data of 95 late FGR and of 154 control group (Fig. 1). All participants gave their written informed consent. For both groups personal and obstetric anamnesis were collected.

The set of examinations (ultrasound-based measures of fetal growth, Doppler measures, cCTG) included in the proposed integrated fetal testing was repeated at fixed timepoints for both cases and controls. In particular, the severity of the growth restriction was assessed by ultrasound biometry every 2 weeks, fetal Doppler velocimetry of UA and MCA and cCTG monitoring weekly from 32 weeks until 36 weeks and twice a week from 36 weeks to delivery; only the last cCTG recording within 24 h of delivery was considered and matched with the last fetal Doppler evaluation within 7 days from delivery. These measurements were done for routine follow-up in late FGR and only for study purposes in the control group.

### Signal acquisition

The antepartum Doppler velocimetry was performed using a Voluson E8 (General Electric Healthcare Technologies, Milwaukee, WI, USA) ultrasound machine equipped with a 2–8 MHz transabdominal transducer. Color Doppler imaging was used to identify the UA and MCA vessels. Doppler flow spectra were obtained from the UA at the midsection of the umbilical cord and the distal portion of the MCA by methods that have been previously described [7].

The antepartum cCTG monitoring was performed in a controlled clinical environment with the patient lying on an armchair. The cCTG records were obtained using Philips Avalon FM30, equipped with an ultrasound transducer and a transabdominal tocodynamometer and lasted at least 60 min. These fetal monitors use an autocorrelation technique to compare the demodulated Doppler signal of a heartbeat with the next one, in order to obtain the heart period (the equivalent of RR period). The resulting heart period is then converted into a heart frequency in beats per minute as soon as a new heart event is detected and accepted. Philips monitors produce a FHR value in bpm every 250 ms (4 values/second.).

In our study the FHR signal was collected simultaneously employing two computerized systems namely, the Philips IntelliSpace Perinatal (www.philips.com/IntelliSpacePerinatal) and the 2CTG2 [16] systems. The Philips IntelliSpace Perinatal uses the NICHD guidelines, and the Oxford system criteria only for Europe. The 2CTG2 systems computes a set of linear and non-linear FHR parameters developed by clinicians [17] and biomedical engineers [18]. The first system is employed for routine antenatal monitoring while the latter provides in addition advanced non-conventional FHR indices for research purposes.

The cardiotocograph was connected to the Philips IntelliSpace Perinatal and the recording is then transferred to the 2CTG2 system [16], which reads both FHR and toco signals two times per second. This frequency represents a reasonable compromise to achieve enough bandwidth and acceptable accuracy for an advanced analysis including non-linear parameters [18]. Each CTG recording was divided in subintervals of either 120 (60 s) and 360 points (180 s), after noise and artifacts removal. The use of 1-min or 3-min subintervals depends on the processing for extracting the different parameters as explained in the next section.

### FHR analysis

A set of parameters, related to the morphology of the signal, according to the standard CTG analysis, were computed: baseline, large and small accelerations, decelerations and contractions per hour. This set includes also time domain measures: FHR mean over 1 min, FHR std., Delta FHR in beats/minute, Short Term Variability (STV), Long Term Variability (LTV) in milliseconds, Interval Index (II) as Arduini et al. reported [17]. Delta Index is the difference between the maximum and the minimum FHR value, excluding acceleration and deceleration from the calculation. Interval Index (II) is calculated as the standard deviation of FHR variations each 2.5 s, normalized by the STV, both computed in 1 min of FHR recording [17].

Moreover, the 2CTG2 software allows computing frequency domain and regularity/nonlinear parameters, which aim at connecting Fetal Heart Rate Variability (FHRV) behavior to physiological mechanisms controlling the heart [18]. Power Spectral Density, estimated by periodogram (direct Fast Fourier Transform, FFT), provide frequency components in specific bands with their associated power: (Low Frequency, LF; Movement Frequency, MF; High Frequency, HF; LF/ (HF + MF) ratio). Approximate Entropy (ApEn) quantifies regularity and complexity of the FHR time series. It measures, indirectly, the correlation and the persistence of a pattern: small values indicate reduced signal irregularity. We use the original definition by Pincus (1995) [19], which demonstrated its usefulness in heart rate analysis [20].

In order to obtain a reliable estimation for these parameters, they were calculated on 3 min subintervals (360 points).

Phase Rectified Signal Average (PRSA) [21] consists in the detection and the quantification of quasiperiodic oscillations in nonstationary signals compromised by noise and artifacts, by synchronizing the phase of all the periodic components. From PRSA we derived the Acceleration Phase Rectified Slope (APRS) and the Deceleration Phase Rectified Slope (DPRS) as suggested in [22]. These parameters are respectively global descriptors of rate of increase (APRS) and rate of decrease (DPRS) of the FHR time series. The method demonstrated its usefulness in providing a global evaluation of the capacity of the heart to rapidly increase and decrease its frequency both in adults and fetuses [23]. As it is known, these events have functional relationships with fetal conditions, both in wellbeing and pathology.

### Statistical analysis

All parameters, except APRS and DPRS which are global values, were averaged on the whole FHR tracing in order to obtain a single parameter value for each recording.

Statistical analyses were performed using SPSS version 23.0. *t*-test was applied for continuous variables whereas chi-square test was used for categorical variables. ANOVA test investigated the existence of association between the tested cCTG features and the outcome (FGR or Control group). *p* value < 0.05 represents the statistical significance for all the tested differences.

## Results

The Kolmogorov-Smirnov test showed a Gaussian distribution in both populations for all parameters investigated. Pregnancy and perinatal outcome are summarized in Tables 1, 2 and 3.

**Table 1:** Differences in pregnancy characteristics between Late FGR and Control group fetuses

Pregnancy characteristics	Late FGR (***n*** = 94) (mean ± SD)	Control group (***n*** = 154) (mean ± SD)	***p-value***
Age^a^ (ys)	32.25 ± 5.23	32.32 ± 5.92	NS
BMI	29.29 ± 4.58	30.25 ± 6.07	NS
Smoker^b^ (%)	8.4	7.3	NS
GA at delivery (wks)	38.4 ± 0.91	39.3 ± 1.18	< 0.001*
Spontaneous delivery (%)	28.7 (27/94)	57.8 (89/154)	**< 0.001**
Cesarean section (%)	69.1 (65/94)	42.2 (65/154)	**< 0.001**

*FGR* Fetal Growth Restriction, *BMI* Body Mass Index, *GA* Gestational Age

^a^T-student test for continuous variables

^b^Chi-square test for categorical variables (values are expressed as a percentage)

^*^values in bold are statistically significant

**Table 2:** Differences in Doppler and cCTG characteristics between Late FGR and Control group fetuses

Doppler and cCTG characteristics	Late FGR (***n*** = 94) (mean ± SD)	Control group (***n*** = 154) (mean ± SD)	***p-value***
UA_PI^a^	1.08 ± 0.42	0.99 ± 0.27	NS
MCA_PI	1.24 ± 0.27	1.44 ± 0.18	0.03*
FHR (bpm)	136.03 ± 10.17	136.68 ± 8.27	NS
STV (ms)	5.90 ± 1.88	6.56 ± 1.75	**< 0.006**
LTV (ms)	19.64 ± 5.04	21.86 ± 4.91	**0.001**
Delta (ms)	37.42 ± 9.51	41.30 ± 8.70	**0.001**
II	0.84 ± 0.05	0.85 ± 0.05	NS
ApEn	1.29 ± 0.14	1.33 ± 0.17	**0.03**
LF (ms^2^)	81.62 ± 5.45	83.12 ± 4.10	**0.01**
MF (ms^2^)	13.1 ± 3.48	12.20 ± 3.03	**0.03**
HF (ms^2^)	5.29 ± 3.80	4.68 ± 2.20	NS
LF/(HF + MF)	4.10 ± 2.12	4.13 ± 2.07	NS
APRS (bpm)	0.36 ± 0.11	0.42 ± 0.10	**< 0.001**
DPRS (bpm)	−0.37 ± 0.12	− 0.45 ± 2.11	**< 0.001**

*FHR* Fetal Heart Rate, *STV* Short Term Variability, *LTV* Long Term Irregularity, *Delta* Delta Index, *II* Interval Index, *ApEn* Approximate Entropy, *LF* Low Frequency, *MF* Movement Frequency, *HF* High Frequency, *LF/(HF + MF)* LF/(HF + MF) ratio, *APRS* Acceleration Phase Rectified Slope, *DPRS* Deceleration Phase Rectified Slope

^a^T-student test for continuous variables

^*^values in bold are statistically significant

**Table 3:** Differences in neonatal outcome between Late FGR and Control group fetuses

Neonatal outcome	Late FGR (***n*** = 94) (mean ± SD)	Control group (***n*** = 154) (mean ± SD)	***p-value***
Newborn weight^a^ (gr.)	2083.79 ± 314.90	3315.65 ± 377.09	< 0.001*
Male^b^ (%)	46.8	55.2	NS
UA pH < 7.05 (%)	0	0	**NS**
UA pCO2	48.18 ± 8.82	45.92 ± 9.39	0.09
UA pO2	19.55 ± 11.53	20.45 ± 9.20	0.58
BE (ecf)	−4.05 ± 3.13	−1.78 ± 2.61	**< 0.001**
Lactates	3.03 ± 1.27	2.11 ± 1.09	**< 0.001**
Apgar 1 min < 7 (%)	0	0	**NS**
Apgar 5 min < 7 (%)	0	0	**NS**
NICU (%)	38.8 (25/94)	5.1 (8/154)	**< 0.001**

*BE* Base Excess, *NICU* Neonatal Intensive Care Unit

^a^T-student test for continuous variables

^b^Chi-square test for categorical variables (values are expressed as a percentage)

^*^values in bold are statistically significant

Before discussing the novel approach dealing with cCTG parameters, we replicated previously reported group characteristic. Pregnant women had similar age, BMI and smoker habits in both control group and late FGR groups. Gestational age at birth occurs on average a week earlier in late FGR and more frequently with cesarean section than control group (Table 1).

No difference in UA_PI values were found between the two groups, while lower MCA_PI values were found comparing late FGR and Control group. Higher UA-PI indices were found only in few late-onset FGRs, while lower MCA-PI indices were showed in most of late-onset cases. CPR was <5Th centile in 39% of IUGR fetus (37/94) while a CPR > 5th centile was observed in all fetuses of the control group.

Among the cCTG time domain parameters, Delta, Short- and Long-Term Variability values exhibited a significant decrease in late FGR compared to Control group fetuses. The more advanced cCTG parameters such as Approximate Entropy (ApEn), Low Frequency (LF) and Movement Frequency (MF) of the power spectral density (PSD) of the CTG signal were significantly different comparing the two groups. Interesting results were obtained employing the novel Phase Rectified Signal Averaging (PRSA) parameters. In fact, both APRS and DPRS were significantly different comparing the two groups (APRS is greater and DPRS is lower in Control group fetuses (Table 2).

The percentage of NICU admission in late FGR was significantly higher than in the Control group.

No difference in newborn sex was observed and a stratified analysis according to newborn sex showed no differences (Table 3).

Indications for delivery in the FGR group are described in Table 4. 12/94 (12.7%) had a spontaneous onset of labor, but 3 of them underwent an urgent c-section for pathological CTG in labor; 53/94 (56%) had a planned cesarean section for cCTG indication and/or EFW <3rd centile and/or Doppler anomalies. 27/94 (28%) underwent induction of labor for fetal growth or Doppler anomalies, of them 5 had a failed induction and 4 underwent an urgent cesarean section for pathological CTG in labor.

**Table 4:** Indication for delivery in FGR group

	cCTG anomalies	Fetal growth/EWF	Doppler anomalies
**Planned C- section (main indication)**	15/94 (16%)	20/94 (21.2%)	18/94 (19%)
**Induction of labor**	–	20/94 (21%)	7/94 (7.4%)
**Spontaneous onset of labor**	–	5/94 (5.3%)	7/94 (7.2%)

Late FGRs were divided in two groups according to the Doppler UA_PI (<95th vs >95th centile), the MCA_PI (>5th vs <5th centile) profiles and the CPR (>5th vs <5th centile) [24]. cCTG parameters were compared between the two groups and the most significant differences were reported in the Table 5. Respect to UA_PI values, the mean values of the cCTG parameters were significantly lower for STV and DPRS when UA_PI was >95th centile, whereas for Delta and APRS the difference was not significant. Respect to MCA_PI values, the mean of all cCTG parameters was significantly lower when ACM_PI was <5th centile. Our results showed that only 28.7% of all late FGRs had UA_PI values >95th centile, while 86.1% of them had MCA_PI values <5th centile. All cCTG parameters investigated exhibited significant changes in cases where MCA_PI values were < 5th centile compared to cases where MCA_PI values >5th centile, while in cases where UA_PI values was >95th centile respect to UA_PI <95th centile only STV and DPRS parameters were different. No significance was found comparing CPR superior to 5th centile versus inferior to 5th centile in the late FGR group.

**Table 5:** Differences in mean values of cCTG parameters with respect to Doppler abnormalities in late FGR

cCTG	UA_PI			MCA_PI			CPR		
	< 95th centile (***n*** = 67) (mean ± SD)	> 95th centile (***n*** = 27) (mean ± SD)	***p*** value	> 5th centile (***n*** = 13) (mean ± SD)	< 5th centile (***n*** = 81) (mean ± SD)	***p*** value	> 5th centile (***n*** = 57) (mean ± SD)	< 5th centile (***n*** = 37) (mean ± SD)	***p*** value
**STV**	6.29 ± 2.12	4.93 ± 1.47	0.03*	6.17 ± 2.00	4.27 ± 1.48	**0.02**	6.03 ± 2.06	5.68 ± 2.03	NS
**Delta**	39.02 ± 10.67	32.79 ± 7.11	NS	38.62 ± 9.71	28.99 ± 9.06	**0.02**	37.64 ± 10.20	36.61 ± 10.23	NS
**APRS**	0.37 ± 0.12	0.31 ± 0.09	NS	0.37 ± 0.11	0.27 ± 0.09	**0.04**	0.35 ± 0.11	0.34 ± 0.12	NS
**DPRS**	− 0.39 ± 0.14	− 0.30 ± 0.09	**0.03**	−0.38 ± 0.14	−0.26 ± 0.09	**0.03**	−0.37 ± 0.13	−0.35 ± 0.14	NS

*STV* Short Term Variability, *Delta* Delta Index, *APRS* Acceleration Phase Rectified Slope, *DPRS* Deceleration Phase Rectified Slope

*values in bold are statistically significant

Moreover, to give further strength to the correlation between cCTG parameters and Doppler indices and to define a cut-off value to distinguish between Control group and late FGR, Doppler indices were predicted from cCTG parameters.

**Fig. 2:**
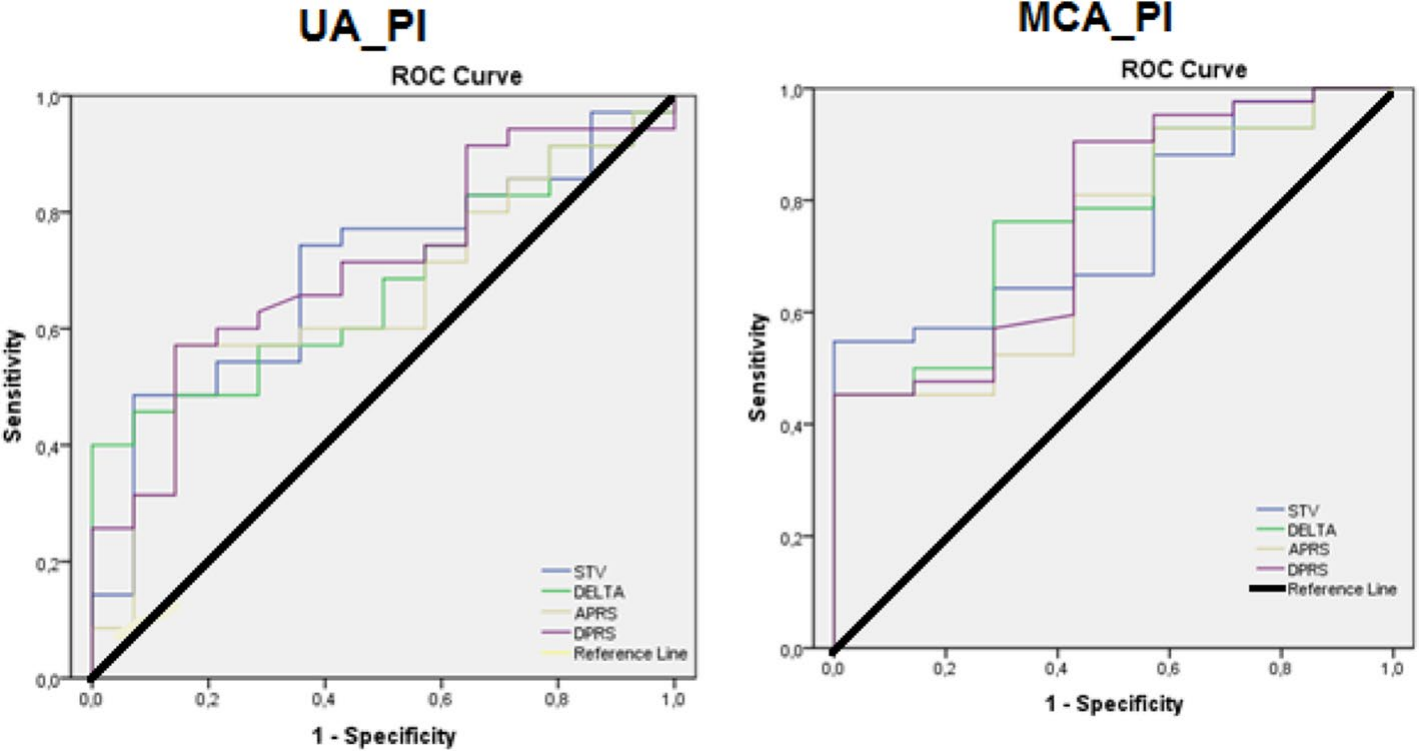
ROC curves for STV, Delta, APRS and DPRS for the binary classification of UA_PI >95th centile (left) and MCA_PI <5th centile (right), respectively. The AUC of ROC curves was accurate for STV (0.76), Delta (0.77), APRS (0.73) and DPRS (0.76) when MCA_PI values were < 5th centile with a cut-off value for STV of 5.24 (sensitivity 0.64 and specificity 0.71), Delta of 32.40 (sensitivity 0.76 and specificity 0.71), APRS of 0.27 (sensitivity 0.81 and specificity 0.57) and DPRS of − 0.24 (sensitivity 0.91 and specificity 0.57)

Therefore, ROC curves were obtained for most significant cCTG parameters respect to Doppler indices. The AUC of ROC curves were accurate for STV (0.70), Delta (0.68), APRS (0.65) and DPRS (0.71) when UA_PI values were > 95th centile. Evaluating the correlation of the same cCTG parameters investigated in cases where MCA-PI values were < 5th centile, the AUC of ROC curves were more accurate for STV (0.76), Delta (0.77), APRS (0.73) and DPRS (0.76) than those obtained when UA_PI were > 95th centile (Fig. 2).

Neonates were more likely admitted to NICU if they had UA_PI >95th percentile (χ^2^ 7,5; *p* < 0.05), MCA_PI <5Th percentile (χ^2^ 4,8; *p* < 0.05), EFW < 2000 g (χ^2^ 40,1 *p* < 0.01).

Considering cCTG parameters, FGR neonates admitted to NICU had lower values of STV (5.2 ± 1.8 vs 6.1 ± 1.8; *p* = 0.02), APRS (0.31 ± 0.10 vs 0.37 ± 0.10; *p* = 0.02), DPRS (− 0.31 ± 0.11 vs − 0.38 ± 0.12; *p* = 0.01) and Delta (34.17 ± 9.94 vs 38.57 ± 9.14 *p* = 0.03) compared to FGR neonates that were not admitted to NICU.

No data were available on the specific indication for NICU admission, on the length of hospitalization and on the short- and long- term neonatal outcomes.

## Discussion

Our study shows that late FGRs with abnormal fetal Doppler values have a significant decrease of several cCTG parameters. These findings are more evident when late FGRs with abnormal MCA_PI indices are assessed. Our results confirm the importance of the integration between MCA_PI Doppler assessment and cCTG parameters, in order to identify more accurately the clinical deterioration of late FGRs and show a potential role of advanced cCTG parameters and Doppler in predicting the perinatal outcome.

These results are in agreement with the existing literature on fetal monitoring. In fact, heart control is less reactive in late FGRs than in control group ones. According to some studies [25, 26], FGRs had a lower cardiac acceleratory and deceleratory capacity respect to control group ones. This reduced reactivity causes lower values of short- and medium-term FHR variability and also a reduced number of increase and decrease of FHR signal.

Therefore, the identification of lower cCTG-derived indices could indicate a less reactive cardiac control and finally could allow the identification of late FGR at risk of adverse outcome.

In the context of longitudinal monitoring, UA_PI deteriorates only in a few cases while MCA_PI has been suggested to be more specific to detect fetuses at risk for adverse outcome [27, 28]. However, a reduction in MCA-PI could indicate an advanced stage of increased brain blood flow, which it is not an entirely protective mechanism. In fact, also mild degrees of hypoxia can induce permanent adaptive changes in the developing brain, which in turn could have effects on neurocognitive development at 2 years of age [29].

To date, arterial Doppler anomalies identify the initial phase of fetal deterioration, before circulatory and biophysical adaptation occurs, and predominantly could be evident on cCTG analysis [10]. Therefore, as Doppler and biophysical anomalies are very subtle, while fetal deterioration can occur very quickly even in term fetuses, the introduction of new cCTG parameters can help the management of late FGR at risk of adverse outcome.

Clinical evolution of late onset FGR is associated with moderate placental insufficiency which has two direct effects on fetal cardiovascular development. First, reduced oxygen and nutrients supply and second, leads to slight increasing of placental resistance and chronic cardiac afterload [30, 31]. In most of the cases villous hypoplasia/thrombosis do not have a significant extent in order to increase the UA PI [32]. Therefore, the first hemodynamic alteration in the presence of hypoxia is the cerebral vasodilatation [33]. Vasodilatation itself is a method of neuroprotection, but it cannot completely compensate the effects of hypoxia, which rapidly causes severe brain injury without the classical sequence of Doppler modifications and cardiac insufficiency, and severe CTG alterations [32]. In fact, several recent studies [4, 12, 32] confirms that cerebral vascular modifications in late FGR are associated with adverse perinatal outcome and potential long-term neurological sequela.

Previous studies [11, 34], have demonstrated that even if late FGR are not affected by prematurity, they had increased brain oxygen requirements and difficulty to tolerate hypoxia [35].

When a CTG trace is reassuring, according to the Dawes and Redman criteria, it could exclude hypoxemia, while a non-reassuring CTG is associated with a wide range of pH values at birth [36]. However, computerized analysis of CTG has shown the opportunity to provide a standardized method to evaluate quantitative measures of FHR variability, which could improve the prediction of adverse perinatal outcome in FGRs [12].

A study published by Crimmins et al. in 2014 [37] demonstrated that > 34 weeks of gestation, stillbirths occur after MCA brain-sparing in a shorter interval than predicted by a normal BPS. Recognition of these differences in clinical behavior requires consideration for the planning of monitoring intervals in term fetal growth restriction. The use of cCTG parameters like PRSA analysis can improve the identification of fetuses whose clinical condition are progressively deteriorating and differently from ultrasound measures, are not operator dependent. FGR children with evidence of fetal circulatory redistribution (preferential perfusion of the brain) had more severe neurodevelopmental impairments than those born FGR alone [38], so the right identification of fetuses who have blood redistribution is important both for in utero mortality and for the future neurodevelopment of the offspring.

The association between MCA_PI and PRSA analysis was demonstrated in a previous study by Stampalija et al. [39], but in our study we selected a cohort of late FGR only.

Our study is a single-centre trial was based on a direct evaluation of all aspects of trial conduct, including data acquisition, quality control, data management, and data analysis. The main limitation of the study resides in the size dimension of the sample and in the fact that the study is retrospective and observational. As a matter of fact, the diagnosis of late FGR, the Doppler results and the cCTG parameters were already known by the clinicians. As a consequence, the late FGR group might underwent an overtreatment, due to the concerns of fetus wellbeing. On the other hand, all decisions on delivery in the FGR group were taken on the basis of cCTG anomalies and/or Doppler measurements indicating fetal deterioration [40]. Another limitation is that no data were available on the specific indication for neonatal admission in NICU, the length of hospitalization and the following short- and long- term neonatal outcomes.

PRSA analysis showed a promising role in identifying the late IUGR fetus. There is some evidence that even SGA fetuses with normal Doppler velocimetry might suffer some degree of growth restriction not identifiable by standard biophysical tools. In this study we showed a correlation between ultrasound Doppler parameters and cCTG parameters, in particular the reduction of MCA_PI was associated with reduction of APRS and DPRS.

In our results we observed an association of UA_PI>95th centile and MCA_PI<5th centile with higher risk for NICU admission, while CPR < 5th centile showed no significance in predicting perinatal outcome according to other studies [41–44]. In the NICU admitted group advanced cCTG parameters, STV, APRS, DPRS and Delta were significantly lower. Those results showed a promising role of advanced cCTG parameters in predicting perinatal outcome that might be further investigated.

New technologies and tools might be helpful in differentiating between SGA and FGR, to identify those fetuses who have a higher demand for antenatal surveillance [40] and to help the clinicians to predict adverse perinatal and neonatal outcome.

## Conclusion

The aim of the study was to verify if an association between Doppler abnormalities and a set of recently proposed cCTG parameters exists in late-onset FGRs.

The results of this study confirm that late FGR requires an integrating fetal surveillance with Doppler vessels and cCTG monitoring, which has an important impact on the clinical follow-up before delivery.

Our results also suggest the contribution of advanced cCTG parameters and fetal Doppler in the identification of late FGR and the associated risk for NICU admission.

Randomized studies are required to evaluate the contribution of cCTG and Doppler parameters, and their integration, in the identification of both short- and long- term neonatal outcomes, to improve the management and timing for delivery in late FGR population.

## Data Availability

The datasets used and/or analyzed during the current study are available from the corresponding author on reasonable request.

## Abbreviations

FGR: Fetal Growth Restriction
IUGR: Intrauterine Growth Restriction
FHR: Fetal Heart Rate
CTG: Cardiotocography
cCTG: Computerized CTG
UA_PI: Umbilical Artery Pulsatility Index
MCA_PI: Middle Cerebral Artery Pulsatility Index
STV: Short Term Variability
LTV: Long Term Variability
Delta: Delta Index
II: Interval Index
ApEn: Approximate Entropy
PSD: Power Spectral Density
LF: Low Frequency
MF: Movement Frequency
HF: High Frequency
LF/(HF + MF): LF/(HF + MF) ratio
APRS: Acceleration Phase Rectified Slope
DPRS: Deceleration Phase Rectified Slope
BMI: Body Mass Index
GA: Gestational Age
BE: Base Excess
NICU: Neonatal Intensive Care Unit
